# Polymicrobial Complexity Rather than Glycemic Control Is Associated with Multidrug Resistance in Diabetic Foot Infections

**DOI:** 10.3390/microorganisms14102401

**Published:** 2026-10-20

**Authors:** Luigi Regenburgh De La Motte, Loredana Deflorio, Francesca Carreras, Michela Salvatici, Chiara Corrado, Lorenzo Drago

**Affiliations:** 1 UOC Laboratory of Clinical Medicine with Specialized Areas, IRCCS MultiMedica, 20138 Milan, Italy; 2 Clinical Microbiology and Microbiome Laboratory, Department of Biomedical Sciences for Health, University of Milan, 20133 Milan, Italy

**Keywords:** diabetic foot infections, multidrug resistance, polymicrobial infections, antimicrobial resistance, glycated hemoglobin, HbA1c

## Abstract

To investigate factors associated with multidrug resistance (MDR) in diabetes-related foot infections (DFIs), focusing on polymicrobial infection and glycated hemoglobin (HbA1c). This retrospective single-center study included culture-positive deep-tissue biopsy specimens collected during 2025. Consecutive positive biopsies from the same patient were grouped into episodes when successive samples were obtained ≤7 days apart. MDR was assessed using organism-specific antimicrobial categories, counting resistant results and excluding expected resistance phenotypes. Logistic regression used patient-clustered robust standard errors. HbA1c was evaluated in a secondary model restricted to measurements within ±30 days of sampling. The 191 culture-positive biopsies yielded 175 episodes from 150 patients; 43 episodes (24.6%) were polymicrobial. The most frequently recovered species were *Staphylococcus aureus* (35.4% of episodes), *Pseudomonas aeruginosa* (20.6%) and *Enterococcus faecalis* (14.9%). Among 157 MDR-evaluable episodes, 49 (31.2%) were MDR-positive. MDR occurred in 46.5% of polymicrobial versus 25.4% of monomicrobial episodes (crude OR 2.55; *p* = 0.020). Polymicrobial infection remained associated with MDR after adjustment for Gram-negative involvement, age and sex (aOR 4.52, 95% CI 1.91–10.72). In the HbA1c-adjusted model (94 episodes; 85 patients), polymicrobial infection remained associated with MDR (aOR 4.04, 95% CI 1.38–11.84), whereas HbA1c was not significantly associated with MDR (aOR 0.91 per 1% increase, 95% CI 0.69–1.20). Polymicrobial infection was associated with MDR, although unmeasured clinical confounding limits interpretation. The HbA1c findings remain inconclusive because of limited precision. These associations require confirmation in larger studies incorporating detailed clinical exposures and wound characteristics.

## 1. Introduction

Diabetic foot infections (DFIs) represent a major clinical and public health challenge, arising as a severe complication of diabetes mellitus, a condition whose global prevalence continues to increase worldwide [1]. DFIs are associated with substantial morbidity, prolonged hospitalization, and high healthcare costs and remain one of the leading causes of non-traumatic lower-limb amputations [2,3,4,5,6,7]. Long-term outcomes are particularly poor, with significant risks of recurrence, limb loss, and mortality over time [4]. From a microbiological perspective, DFIs are complex and heterogeneous infections, frequently involving polymicrobial communities that include aerobic and anaerobic organisms [8,9]. The composition of these infections is influenced by ulcer severity, chronicity, and prior antimicrobial exposure. Conventional culture-based diagnostic approaches may underestimate the true microbial burden and fail to detect fastidious or anaerobic pathogens, thereby limiting diagnostic accuracy [10]. Current international guidelines, including those from the International Working Group on the Diabetic Foot, emphasize the importance of appropriate microbiological sampling and targeted antimicrobial therapy in the management of DFIs [11]. Antimicrobial resistance (AMR) further complicates the management of these infections, contributing to therapeutic failure and adverse outcomes [12,13]. Recent evidence suggests that resistance patterns in DFIs have evolved over time, including significant shifts observed during the COVID-19 pandemic [12,13]. Similar longitudinal changes in pathogen distribution and antimicrobial resistance have also been described in other infectious settings, including respiratory tract infections [14]. These findings highlight the dynamic nature of DFI microbiology and the need for continuous epidemiological surveillance. Glycemic control, commonly assessed through glycated hemoglobin (HbA1c), is a key determinant of infection susceptibility in patients with diabetes. Elevated HbA1c levels have been associated with an increased risk of infections in patients with diabetes [15]. In diabetic foot disease, the relationship between HbA1c and wound healing is less consistent; Fesseha et al. found no clinically meaningful overall association [16]. In parallel, microbiota-oriented studies have shown that DFIs often display complex and polymicrobial microbial communities that may be underestimated by conventional culture-based methods [10,17,18]. However, whether glycemic status is directly associated with microbiological complexity, polymicrobial infection structure, and antimicrobial resistance in DFIs remains insufficiently explored. In this context, the present study aimed to investigate the association between HbA1c levels and microbiological characteristics of DFIs, with a specific focus on polymicrobial organization and antimicrobial resistance patterns.

## 2. Materials and Methods

### 2.1. Study Design and Patient Selection

This retrospective observational study was conducted at the Laboratory of Clinical Medicine with Specialized Areas, IRCCS MultiMedica, Milan, Italy, and included patients with diabetes-related foot infections (DFIs) managed between January and December 2025. The diagnosis of DFI was established during routine clinical care, whereas the present retrospective analysis was based on microbiological and laboratory records and did not independently re-adjudicate infection status from clinical signs and symptoms. Microbiological records were retrospectively screened by selecting specimens recorded as biopsies in the laboratory database. All included specimens were deep-tissue biopsies collected during routine clinical care and submitted for bacterial culture. Superficial swab specimens were excluded. Detailed information on the sampling technique and adherence to a standardized collection protocol was not available in the retrospective laboratory dataset. The initial microbiological dataset comprised 3348 records corresponding to 419 unique biopsy specimens. Of these, 191 biopsies yielded bacterial growth and were included in the construction of infection episodes.

**Figure 1:**
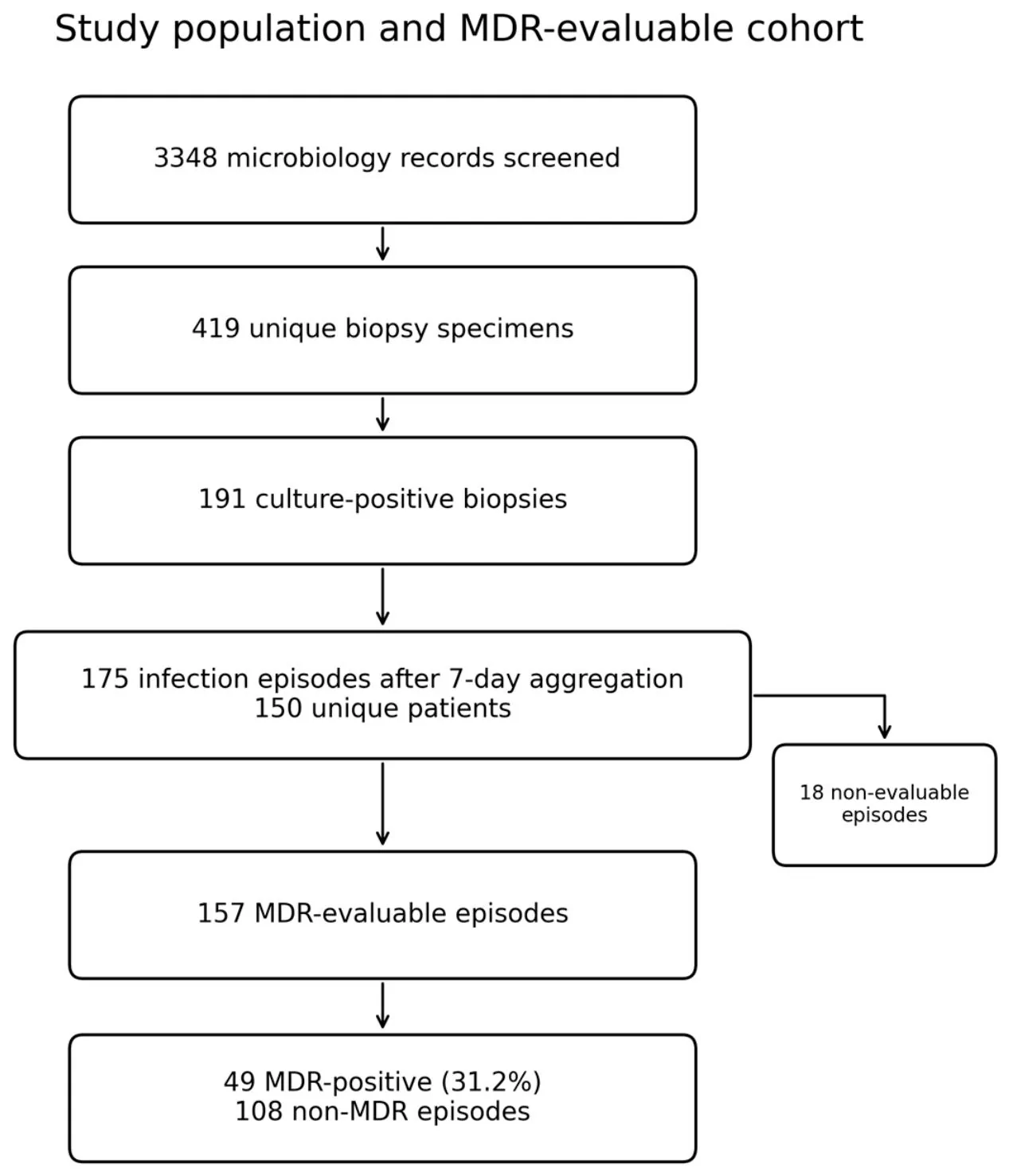
Study population and construction of the MDR-evaluable cohort. A total of 3348 microbiology records corresponding to 419 unique deep-tissue biopsy samples were screened. Among 191 culture-positive biopsies, aggregation of consecutive positive samples from the same patient within 7 days resulted in 175 infection episodes from 150 patients. Of these, 157 episodes were evaluable according to the standardized MDR definition, and 18 were non-evaluable. Forty-nine of the 157 evaluable episodes (31.2%) were MDR-positive.

Consecutive culture-positive biopsies from the same patient were grouped into a single infection episode when the interval between successive positive biopsies was ≤7 days. A new episode was defined when this interval exceeded 7 days. The date of the first positive biopsy was considered the episode index date, and all distinct bacterial species recovered from biopsies belonging to the same episode were combined to define its microbiological composition. This procedure resulted in 175 culture-positive infection episodes from 150 individual patients (Figure 1; Table 1). Because some patients contributed more than one temporally distinct episode, within-patient clustering was accounted for in the multivariable analyses. HbA1c measurements were retrospectively retrieved from laboratory records and linked to infection episodes when available.

### 2.2. Microbiological Analysis and Antimicrobial Susceptibility Testing

Microbiological analyses were performed according to routine standardized laboratory procedures. Bacterial isolates were identified using the Autof MS1000 matrix-assisted laser desorption ionization–time of flight mass spectrometry (MALDI-TOF MS) system (Autobio Diagnostics Co., Ltd., Zhengzhou, China). In routine laboratory practice, identification results with scores ≥ 8 were considered acceptable when consistent with culture purity and colony morphology. Low-confidence or inconclusive results were reassessed after checking culture purity, and MALDI-TOF MS acquisition was repeated in manual mode by selecting an alternative area of the bacterial deposit within the same target spot. Antimicrobial susceptibility testing (AST) was subsequently performed using the VITEK^®^ 2 system (bioMérieux, Marcy-l’Étoile, France), and susceptibility categories were interpreted according to the EUCAST clinical breakpoints applicable at the time of testing. Routine quality-control procedures included reference strains *Escherichia coli* ATCC 25922, *Staphylococcus aureus* ATCC 29213 and *Pseudomonas aeruginosa* ATCC 27853.

### 2.3. Definition of Infection Episodes and Polymicrobial Infection

The infection episode was used as the primary unit of analysis. Episodes containing one bacterial species were classified as monomicrobial, whereas polymicrobial infection was defined as recovery of two or more distinct bacterial species within the same episode. When more than one culture-positive biopsy belonged to the same 7-day episode, species recovered across all biopsies were combined, and duplicate recovery of the same species was counted only once for the purpose of defining episode-level microbial composition.

### 2.4. Definition of Multidrug Resistance

Multidrug resistance (MDR) was assessed initially at the isolate level using the standardized organism-specific antimicrobial categories proposed by Magiorakos et al. MDR was defined as acquired resistance to at least one antimicrobial agent in three or more relevant antimicrobial categories [19]. Because antimicrobial susceptibility results in the present cohort were generated using contemporary EUCAST interpretation, only isolates categorized as resistant (R) were considered resistant for the primary MDR analysis. Isolates categorized as susceptible, increased exposure (I), were not combined with resistant isolates, in accordance with the current EUCAST definitions of susceptibility categories. Expected or intrinsic resistance phenotypes were excluded from the calculation of acquired resistance. MDR classification was restricted to bacterial groups for which standardized organism-specific MDR criteria could be applied, including *S. aureus*, *Enterococcus* spp., *Enterobacterales* and *P. aeruginosa*. Episodes containing exclusively organisms for which a standardized MDR classification could not be assigned were considered non-evaluable for the primary MDR endpoint. Of the 175 infection episodes, 157 were MDR-evaluable and 18 were classified as non-evaluable. For *S. aureus*, methicillin-resistant *S. aureus* (MRSA) isolates were classified as MDR in accordance with the standardized MDR framework, which considers methicillin resistance to confer resistance across the relevant β-lactam categories. Newer β-lactam/β-lactamase inhibitor combinations not included in the original standardized framework were not considered as additional independent antimicrobial categories in the primary analysis. At the episode level, an MDR-positive episode was defined by the presence of at least one MDR isolate among the evaluable organisms recovered during that episode.

In the Magiorakos framework, MDR denotes non-susceptibility to at least one agent in three or more organism-specific antimicrobial categories; XDR denotes non-susceptibility to at least one agent in all but two or fewer categories, and PDR denotes non-susceptibility to all agents in all relevant categories. The present MDR analysis used the R-only adaptation described above. XDR and PDR classifications were not assigned because the available susceptibility panels did not comprehensively cover the required agents and categories. Resistance phenotypes were additionally described using available AST results: MRSA was identified by oxacillin resistance, VRE by vancomycin resistance, and carbapenem resistance by meropenem resistance. Results for carbapenem/β-lactamase inhibitor combinations were not used as substitutes for meropenem alone. These phenotypic findings do not establish the underlying resistance mechanisms.

### 2.5. Glycemic Assessment

HbA1c was investigated as a secondary clinical variable. For each infection episode, all available HbA1c measurements from the same patient in the source laboratory export were reviewed. The measurement closest in absolute elapsed time to the index biopsy was selected; an exact temporal tie was resolved by selecting the earlier measurement. The primary HbA1c-adjusted analysis was restricted to selected measurements within 30 days before or after the index biopsy. This study-specific window limited temporal separation between glycemic assessment and microbiological sampling and was not a guideline-defined interval. Values recorded in mmol/mol were converted to percentages using HbA1c (%) = 0.09148 × HbA1c (mmol/mol) + 2.152. HbA1c was analyzed continuously in regression models. Descriptive categories of <7.0%, 7.0–7.9% and ≥8.0% were used with reference to commonly used treatment targets. An HbA1c target <7.0% is appropriate for many nonpregnant adults, whereas less stringent targets may be appropriate according to individual circumstances [20]. These categories did not classify achievement of individualized goals or define MDR-risk thresholds. HbA1c linkage was verified against the source records. Two exploratory sensitivity analyses used the following rules: (i) all episodes with a selected HbA1c measurement, without an additional temporal restriction; and (ii) the subset whose selected measurement was obtained on or before the calendar date of the index biopsy. The second analysis did not replace a selected post-index value with another preceding measurement. These sensitivity analyses were exploratory.

### 2.6. Statistical Analysis

Analyses were performed at the infection-episode level. Categorical variables were summarized as counts and percentages, and continuous variables, using appropriate measures of central tendency and dispersion according to their distribution. Differences in categorical variables were assessed using Fisher’s exact test or the χ^2^ test, as appropriate. The crude association between polymicrobial infection and MDR was assessed using Fisher’s exact test and expressed as an odds ratio (OR) with a 95% confidence interval (CI). Multivariable logistic regression was used to investigate factors independently associated with MDR positivity. Because individual patients could contribute more than one infection episode, regression models used patient-clustered robust standard errors to account for within-patient correlation. The primary model included polymicrobial infection, Gram-negative involvement, age and sex as prespecified covariates. A secondary model conducted among episodes with an HbA1c measurement within ±30 days additionally included HbA1c as a continuous covariate. Sensitivity analyses repeated the HbA1c model using alternative temporal definitions of HbA1c availability. Associations are reported as adjusted odds ratios (aORs) with 95% CIs. All tests were two-sided, and a *p*-value < 0.05 was considered statistically significant. Data processing and statistical analyses were performed using Python v.3.12.14 with pandas v.2.2.3, NumPy v.2.3.5, SciPy v.1.17.0 and statsmodels v.0.15.0.

## 3. Results

### 3.1. Study Population and Episode Construction

A total of 3348 microbiology records corresponding to 419 unique deep-tissue biopsy specimens were screened. Bacterial growth was recovered from 191 biopsies. After grouping consecutive culture-positive biopsies obtained from the same patient within 7 days, 175 infection episodes from 150 individual patients were identified (Figure 1; Table 1). Of these, 132 episodes (75.4%) were monomicrobial and 43 (24.6%) were polymicrobial. Overall, 232 episode-level species occurrences representing 23 bacterial taxa were identified. The most frequently recovered organisms at the episode level were *Staphylococcus aureus* (62/175, 35.4%), *Pseudomonas aeruginosa* (36/175, 20.6%), *Enterococcus faecalis* (26/175, 14.9%), *Proteus mirabilis* (20/175, 11.4%), *Enterobacter cloacae complex* (17/175, 9.7%), *Corynebacterium striatum* (13/175, 7.4%), *Escherichia coli* (11/175, 6.3%) and *Klebsiella pneumoniae* ssp. *pneumoniae* (9/175, 5.1%). Because more than one organism could be recovered within an episode, species percentages are not mutually exclusive. Gram-negative organisms were present in 104/175 episodes (59.4%), including 62/132 monomicrobial episodes (47.0%) and 42/43 polymicrobial episodes (97.7%).

### 3.2. Microbial Composition and Co-Occurrence in Polymicrobial Episodes

**Figure 2:**
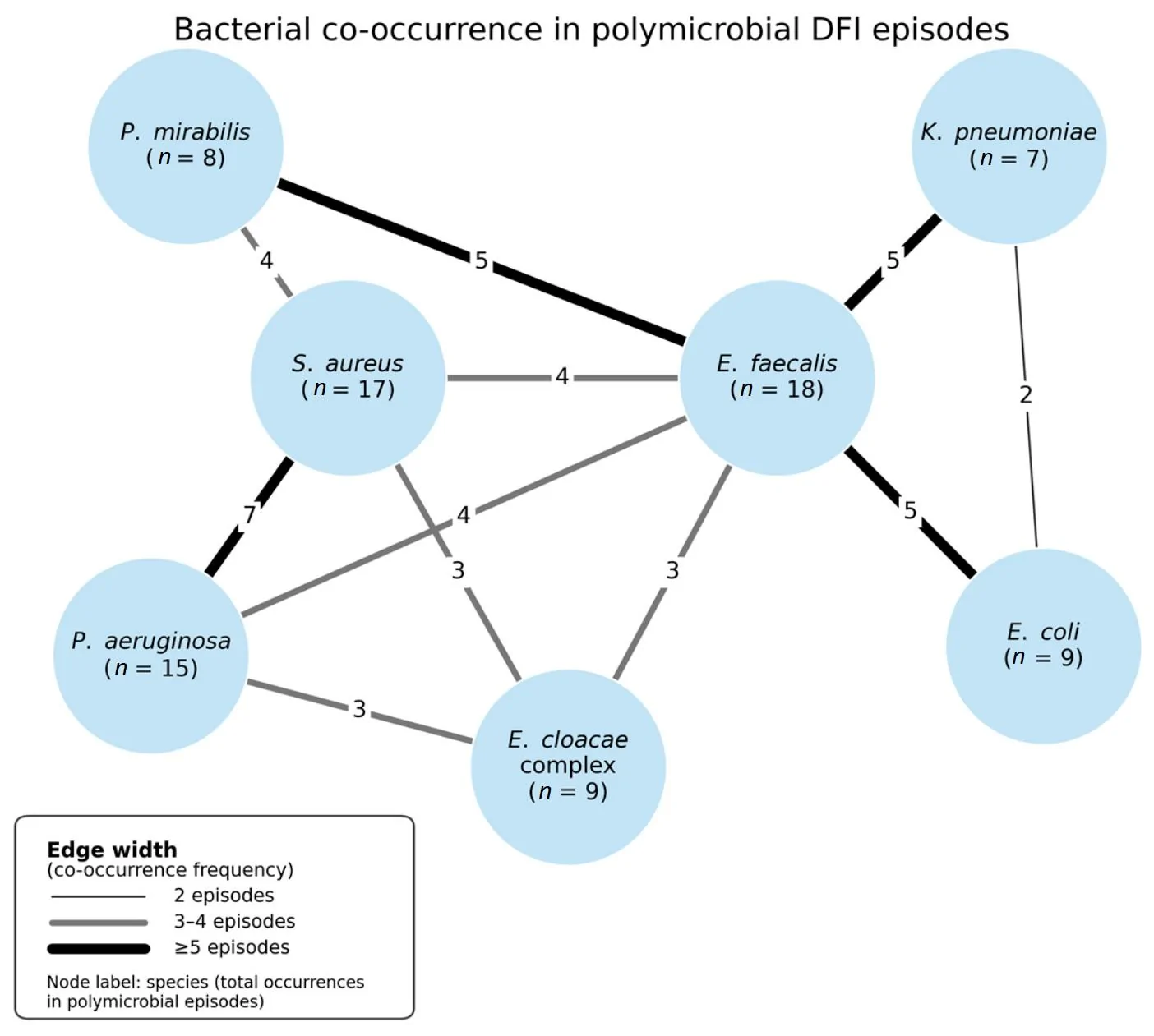
Bacterial co-occurrence network in polymicrobial diabetes-related foot infection episodes. Nodes represent the most frequently recovered bacterial species among the 43 polymicrobial episodes, with node labels indicating the number of episodes in which each species was detected. Edges represent recurrent pairwise co-occurrence, with edge width proportional to the number of shared episodes. Only pairwise co-occurrences observed in at least two episodes are displayed. The network is descriptive and does not imply biological interaction between species.

The 43 polymicrobial episodes comprised 100 episode-level species occurrences: 30 episodes contained two species, 12 contained three species and one contained four species. *E. faecalis* was recovered in 18/43 polymicrobial episodes, *S. aureus* in 17/43, *P. aeruginosa* in 15/43, *E. coli* and *E. cloacae* complex in 9/43 each, *P. mirabilis* in 8/43 and *K. pneumoniae* ssp. *pneumoniae* in 7/43. Recurrent co-occurrence patterns were observed within these episodes (Figure 2). The most frequent pair was *P. aeruginosa* with *S. aureus* (7 episodes). Other recurrent combinations included *E. faecalis* with *P. mirabilis*, *E. coli* or *K. pneumoniae* ssp. *pneumoniae* (5 episodes each); *E. faecalis* with *S. aureus*, *E. faecalis* with *P. aeruginosa*, and *P. mirabilis* with *S. aureus* (4 episodes each); and *E. cloacae* complex with *S. aureus*, *P. aeruginosa* or *E. faecalis* (3 episodes each). These analyses describe observed co-occurrence frequencies and were not used to infer biological interaction between species.

### 3.3. MDR Burden and Organism-Specific Distribution

**Figure 3:**
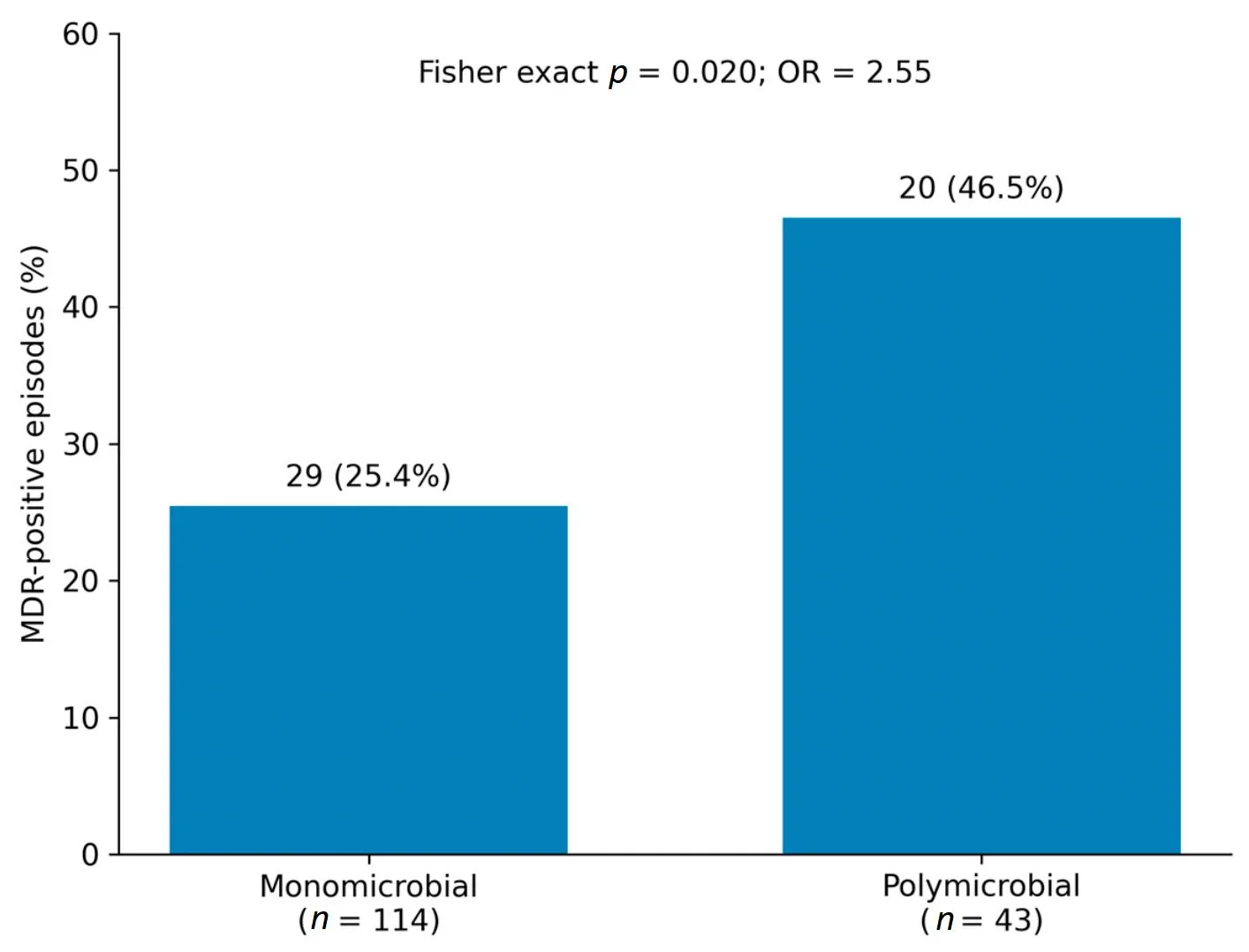
Prevalence of multidrug resistance according to monomicrobial or polymicrobial infection status. MDR was identified in 29/114 (25.4%) evaluable monomicrobial episodes and 20/43 (46.5%) polymicrobial episodes. Polymicrobial episodes had higher crude odds of MDR than monomicrobial episodes (OR 2.55; Fisher’s exact *p* = 0.020).

Of the 175 infection episodes, 157 (89.7%) contained at least one organism evaluable according to the standardized organism-specific MDR framework; 18 episodes (10.3%) were therefore non-evaluable for the primary MDR endpoint. Among the 157 evaluable episodes, 49 (31.2%) were MDR-positive. MDR was detected in 29/114 monomicrobial episodes (25.4%) compared with 20/43 polymicrobial episodes (46.5%), corresponding to a crude OR of 2.55 (Fisher’s exact *p* = 0.020) (Figure 3). Thus, polymicrobial episodes accounted for approximately one quarter of all culture-positive episodes but showed an MDR prevalence almost twice that observed in evaluable monomicrobial episodes. At the isolate level, MDR prevalence was heterogeneous among organisms covered by the standardized framework (Table 2). MDR was identified in 34/67 *S. aureus* isolates (50.7%), 12/22 *P. mirabilis* isolates (54.5%), 9/10 *K. pneumoniae* ssp. *pneumoniae* isolates (90.0%), 4/12 *E. coli* isolates (33.3%), 2/37 *P. aeruginosa* isolates (5.4%) and 1/17 *E. cloacae* complex isolates (5.9%); no *E. faecalis* isolate met the MDR definition (0/27). Phenotypic characterization identified 30/67 *S. aureus* isolates (44.8%) as MRSA and 1/29 enterococcal isolates (3.4%) as VRE; the latter was *E. faecium*. Meropenem resistance was detected in 7/86 Enterobacterales isolates (8.1%), all *K. pneumoniae*. No meropenem-resistant *P. aeruginosa* was detected among the 36 isolates with available results; six were categorized as susceptible, increased exposure (I), and 30 as susceptible (S). One additional *P. aeruginosa* isolate lacked a meropenem result. No *A. baumannii* was recovered. Organisms not covered by the standardized species-specific MDR framework were not assigned an MDR status. Species-level estimates were considered descriptive, particularly where isolate numbers were small, and no formal between-species comparisons were performed.

### 3.4. Multivariable Analysis of Factors Associated with MDR

**Figure 4:**
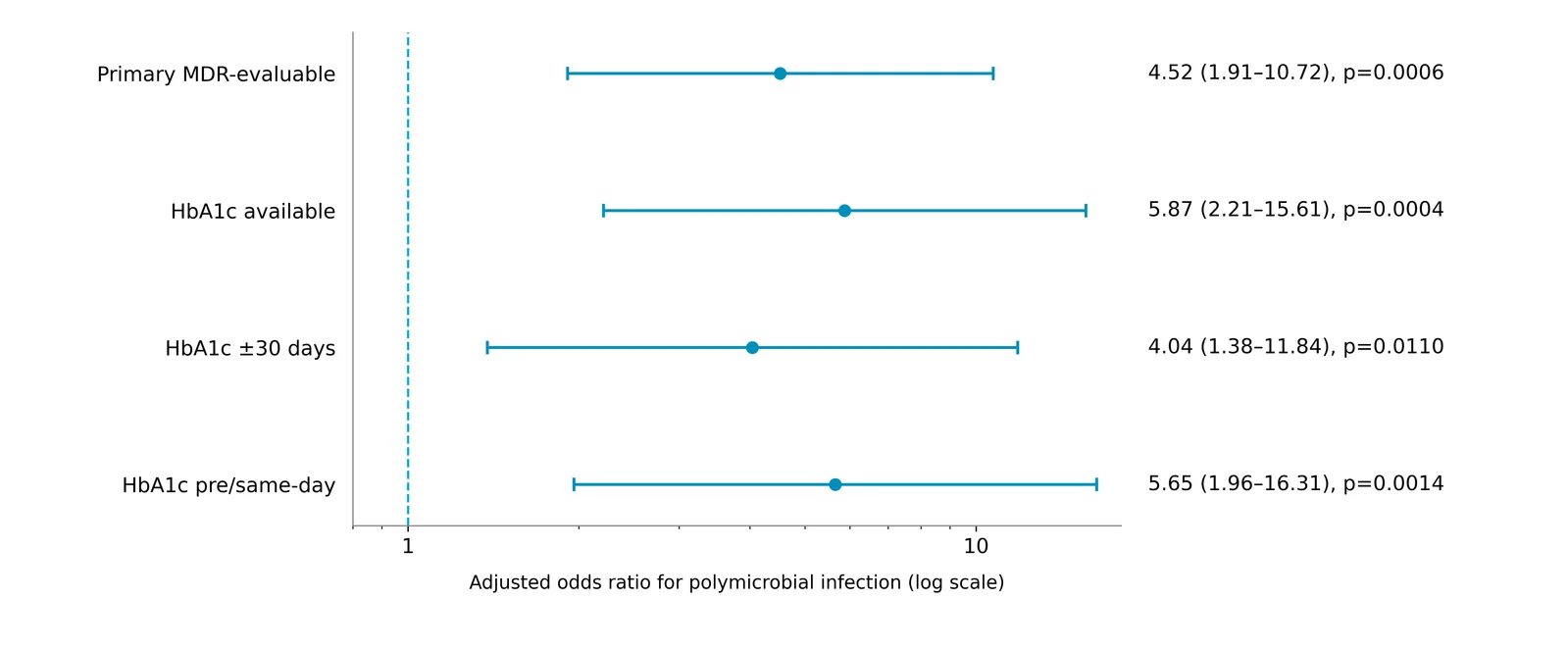
Association between polymicrobial infection and multidrug resistance across multivariable analyses. Points and horizontal lines represent adjusted odds ratios and 95% confidence intervals. The primary model used all 157 MDR-evaluable episodes; the HbA1c ±30-day model used 94 episodes. The reconciled exploratory sensitivity analyses used 124 episodes with any selected HbA1c measurement and 104 episodes whose selected measurement was obtained on or before the index calendar date. All models used patient-clustered robust standard errors and adjusted for Gram-negative involvement, age and sex; HbA1c models additionally included continuous HbA1c. The dashed line indicates an odds ratio of 1.0.

The primary multivariable model included 157 MDR-evaluable episodes from 135 patients and accounted for repeated episodes within patients using patient-clustered robust standard errors. After adjustment for Gram-negative involvement, age and sex, polymicrobial infection remained associated with MDR (aOR 4.52, 95% CI 1.91–10.72; *p* < 0.001). Gram-negative involvement showed an inverse association with MDR (aOR 0.38, 95% CI 0.15–0.96; *p* = 0.042), whereas age (aOR 1.01 per year, 95% CI 0.97–1.05; *p* = 0.536) and male sex (aOR 0.82, 95% CI 0.34–1.97; *p* = 0.656) were not significantly associated with MDR (Table 3; Figure 4). The association between polymicrobial infection and MDR therefore persisted after adjustment for the prespecified demographic and microbiological covariates.

### 3.5. HbA1c and MDR

An HbA1c measurement was available in the source records for 140/175 infection episodes. The selected measurements were obtained before the index calendar date in 102 episodes, on the same date in 13 and after it in 25; the median calendar interval was 7 days before sampling. A total of 107 episodes had a selected measurement within ±30 days. After restriction to MDR-evaluable episodes, 94 episodes from 85 patients entered the primary HbA1c-adjusted model, including 33 MDR-positive episodes. No statistically significant association was detected between HbA1c and MDR (aOR 0.91 per 1% increase, 95% CI 0.69–1.20; *p* = 0.510). Polymicrobial infection remained associated with MDR (aOR 4.04, 95% CI 1.38–11.84; *p* = 0.011). In the reconciled exploratory sensitivity analysis including all MDR-evaluable episodes with an available selected HbA1c measurement (124 episodes from 107 patients), polymicrobial infection remained associated with MDR (aOR 5.87, 95% CI 2.21–15.61; *p* < 0.001), whereas HbA1c was not significantly associated with MDR (aOR 0.87, 95% CI 0.67–1.13; *p* = 0.299). Restriction to episodes whose selected HbA1c measurement was obtained on or before the index calendar date included 104 episodes from 89 patients. Polymicrobial infection remained associated with MDR (aOR 5.65, 95% CI 1.96–16.31; *p* = 0.001), whereas HbA1c was not significantly associated with MDR (aOR 0.87, 95% CI 0.67–1.15; *p* = 0.330). Figure 4 presents the polymicrobial estimates across these analyses.

## 4. Discussion

The present study investigated microbiological factors associated with multidrug resistance in culture-positive diabetes-related foot infection (DFI) episodes, with particular attention to polymicrobial infection and glycemic control. The principal finding was a consistent association between polymicrobial infection and MDR. Among MDR-evaluable episodes, MDR was detected in 46.5% of polymicrobial infections compared with 25.4% of monomicrobial infections, and polymicrobial infection remained independently associated with MDR after adjustment for Gram-negative involvement, age and sex. In contrast, HbA1c was not independently associated with MDR in the prespecified ±30-day analysis or in sensitivity analyses using alternative temporal definitions of HbA1c availability. Together, these findings indicate that, within this cohort, polymicrobial infection was more closely associated with the presence of MDR organisms than contemporaneous glycemic control. The association between polymicrobial infection and MDR represents the most consistent finding of the study. Polymicrobial episodes accounted for approximately one quarter of all culture-positive infection episodes but showed an MDR prevalence almost twice that observed in monomicrobial episodes. Importantly, this association persisted after adjustment for the prespecified demographic and microbiological covariates and remained consistent across the different HbA1c analytical subsets. Polymicrobial involvement is a well-recognized feature of DFIs, particularly when more comprehensive microbiological approaches are applied. Culturomics-based investigation has demonstrated substantial bacterial richness in moderate-to-severe DFIs, with most infections in that cohort containing multiple organisms [18]. The observed association does not establish that polymicrobial infection is a biological determinant of MDR. Polymicrobiality may instead act as a surrogate marker of wound chronicity, previous antimicrobial exposure and healthcare contact. Information on prior antibiotic treatment, hospitalization history, ulcer duration and severity, peripheral arterial disease and previous interventions was unavailable; these factors could confound the association between polymicrobial status and MDR. Accordingly, the adjusted association should be interpreted as conditional on the demographic and microbiological variables included in the model, rather than as evidence of independence from these unmeasured clinical determinants. This distinction is particularly relevant because antimicrobial resistance in DFIs is influenced by previous clinical exposures. In a tertiary-care DFI cohort, Caruso et al. identified previous hospitalization and recent or current antibiotic exposure as factors associated with antibiotic-resistant infections [13]. Furthermore, longitudinal data from our institution have documented substantial temporal variation in the resistance profiles of major DFI pathogens, including declining methicillin resistance among *S. aureus* and carbapenem resistance among *P. aeruginosa*, alongside heterogeneous trends among *Enterobacterales* [12]. These observations emphasize that the relationship between polymicrobial infection and MDR identified in the present study should be interpreted within the broader epidemiological and antimicrobial-exposure context rather than as evidence of a direct causal effect of microbial diversity on resistance. The microbiological composition of polymicrobial episodes provides additional context for the observed association. Gram-negative organisms were recovered from 42 of 43 polymicrobial episodes, compared with less than half of monomicrobial episodes, and several clinically relevant organisms occurred repeatedly in mixed-species infections. *E. faecalis*, *S. aureus* and *P. aeruginosa* were among the most frequently represented organisms, while recurrent combinations included *P. aeruginosa* with *S. aureus* and *E. faecalis* with several *Enterobacterales*. Previous investigations using expanded culture or molecular approaches have demonstrated that conventional culture may incompletely represent the microbial diversity of chronic wounds and DFIs [10,18]. Recent reviews also emphasize the complex microbiology of DFIs, including the contribution of organisms that may be underdetected by routine aerobic culture [9]. Nevertheless, the co-occurrence analysis performed here was descriptive. Repeated recovery of two organisms within the same infection episode cannot establish direct biological interaction, metabolic cooperation, spatial organization or a shared resistance mechanism. Accordingly, the co-occurrence network should be interpreted as a representation of the observed composition of polymicrobial infections rather than evidence of a structured microbial ecosystem. Demonstrating interspecies interactions would require approaches capable of resolving microbial spatial organization, functional activity or experimentally reproducible interactions, which were beyond the scope of the present culture-based study. Species-specific MDR findings further demonstrate that the association between polymicrobial infection and resistance was not attributable to a uniform resistance phenotype across the bacterial population. MDR prevalence varied markedly among evaluable organisms. Species-specific proportions were descriptive and were not intended to support comparisons between organisms. In particular, the finding of MDR in both *E. faecium* isolates (2/2) should not be interpreted as a stable estimate of resistance prevalence. Likewise, the absence of MDR in the single *C. freundii* and *P. hauseri* isolates provides insufficient evidence to characterize resistance in these species. These observations require confirmation in larger samples. High proportions were observed among *K. pneumoniae* and *P. mirabilis*, although these estimates should be interpreted cautiously because of the relatively small species-specific denominators. *S. aureus* represented both a frequently recovered organism and an important contributor to the overall MDR burden, whereas MDR was uncommon among *P. aeruginosa* isolates in this cohort and was not identified among *E. faecalis*. Such heterogeneity is consistent with the marked species- and time-dependent variability of AMR reported in DFI surveillance studies [12]. These findings support the use of organism-specific susceptibility interpretation rather than considering polymicrobial DFIs as having a homogeneous resistance profile. Gram-negative involvement showed an inverse association with MDR in the primary adjusted model (aOR 0.38, 95% CI 0.15–0.96). This finding should not be interpreted as a protective effect. MDR prevalence varied substantially between Gram-negative species: resistance was frequent among *K. pneumoniae* and *P. mirabilis* but uncommon among *P. aeruginosa* in this cohort. Furthermore, Gram-negative involvement overlapped strongly with polymicrobial status: 42 of 43 polymicrobial episodes contained Gram-negative organisms, leaving only one polymicrobial episode without Gram-negative involvement. This limited representation constrains the interpretation of their separate adjusted associations. The inverse association may therefore reflect the species composition and covariate distribution of this cohort and requires confirmation in larger datasets. The analysis of HbA1c provides a complementary clinical perspective. Poor glycemic control is associated with increased susceptibility to infection among people with diabetes [15]. However, the relationship between HbA1c and outcomes specifically related to diabetic foot disease is less straightforward. Fesseha et al. found no clinically meaningful overall association between baseline or prospective HbA1c and wound healing in patients with diabetic foot ulcers, illustrating that HbA1c alone may not capture the multiple determinants of local wound outcome [16]. In the present study, no statistically significant association was detected between HbA1c and MDR. However, the primary HbA1c-adjusted model included only 94 episodes from 85 patients, with 33 MDR-positive episodes and five explanatory variables. The adjusted estimate for HbA1c was 0.91 per 1% increase (95% CI 0.69–1.20), indicating uncertainty compatible with both lower and higher odds of MDR. This result should therefore not be interpreted as demonstrating the absence of an association. The sensitivity analyses also yielded non-significant HbA1c estimates, but their overlapping samples and shared limitations do not resolve this uncertainty. The unrestricted sensitivity analysis also included temporally remote measurements and should not be interpreted as contemporaneous glycemic assessment. Polymicrobial infection remained associated with MDR in the HbA1c-adjusted model, although its confidence interval was wide. Larger studies with more complete HbA1c availability and detailed clinical covariates are needed to estimate these associations more precisely. HbA1c represents systemic glycemic exposure over the preceding months, whereas the acquisition and selection of antimicrobial resistance may be more directly influenced by factors such as previous antimicrobial therapy, hospitalization, chronic wound duration, repeated interventions and local epidemiology. The absence of a statistically significant association between HbA1c and MDR therefore addresses a different question from the established relationship between diabetes control and overall susceptibility to infection. From a clinical microbiology perspective, the present findings reinforce the importance of appropriate sampling and organism-level susceptibility testing in DFIs. Current IWGDF/IDSA guidance recommends that soft-tissue DFI be diagnosed clinically and, when microbiological assessment is indicated, recommends obtaining an aseptically collected tissue specimen by curettage or biopsy rather than relying on superficial sampling [11]. The same guideline supports conventional microbiological techniques as the first-line approach for pathogen identification. The exclusive use of deep-tissue biopsy specimens in the present study therefore represents a methodological strength. Nevertheless, culture findings must remain integrated with the clinical assessment because microbiological recovery alone does not establish infection, and the diagnosis of DFI remains based on clinical evidence of local or systemic inflammation. Several limitations should be acknowledged. First, this was a retrospective single-center study, which limits external generalizability and does not permit causal inference. Second, although DFI had been established during routine clinical care, the retrospective laboratory dataset did not allow independent re-adjudication of infection according to clinical signs, infection severity or detailed wound characteristics. Third, several potentially important confounders were unavailable, including previous antimicrobial exposure, ulcer duration and severity, peripheral arterial disease, previous hospitalization and repeated wound interventions. The relevance of these variables is particularly important given previous evidence linking antibiotic exposure and hospitalization with resistant DFI isolates [13]. Residual confounding therefore cannot be excluded. Fourth, HbA1c was unavailable for a proportion of infection episodes, reducing the sample size of the glycemic analyses and potentially introducing selection bias. Fifth, conventional culture may underestimate fastidious, anaerobic or otherwise difficult-to-culture organisms and does not provide a complete representation of microbial diversity [9,10]. Finally, MDR classification was restricted to bacterial groups for which the standardized organism-specific framework could be appropriately applied. Consequently, 18 episodes were non-evaluable for the primary MDR endpoint. Although this reduced the evaluable sample size, it avoided assigning potentially misleading MDR classifications to organisms not covered by the standardized definitions.

## 5. Conclusions

In this retrospective single-center cohort of culture-positive diabetes-related foot infection episodes, polymicrobial infection was associated with MDR after adjustment for the measured demographic and microbiological covariates. Polymicrobiality may reflect wound chronicity and previous healthcare or antimicrobial exposure rather than a direct biological determinant of resistance. No statistically significant association was detected between HbA1c and MDR, but the limited sample size and precision preclude concluding that no association exists. These findings support interpreting culture and susceptibility results within the clinical context. Larger multicenter studies incorporating antimicrobial exposure, wound characteristics, vascular status and infection severity are needed to assess the reproducibility of these associations.

## Figures and Tables

**Table 1:** Study cohort and distribution of multidrug resistance according to infection episode characteristics.

Primary MDR Results	
MDR episodes	49/157 (31.2%)
Monomicrobial MDR	29/114 (25.4%)
Polymicrobial MDR	20/43 (46.5%)
Fisher exact OR	2.55
Fisher exact *p*	0.02

**Table 2:** Species-specific distribution of multidrug resistance among evaluable bacterial isolates. MDR prevalence was calculated at the isolate level; therefore, isolate counts may exceed the number of infection episodes in which a given species was recovered because multiple isolates of the same species could occur within a single 7-day infection episode. MDR classification was restricted to bacterial groups covered by the standardized organism-specific MDR framework. Organisms not covered by the framework were not assigned an MDR status and are not included. Species-specific estimates are descriptive and should be interpreted cautiously for organisms represented by small numbers of isolates. MDR, multidrug resistance.

Species	Isolates	MDR Isolates	MDR %	Median Resistant Categories
*Citrobacter braakii*	3	0	0.0%	0
*Citrobacter freundii*	1	0	0.0%	1
*Citrobacter koseri*	2	0	0.0%	0.5
*Enterobacter aerogenes*	1	0	0.0%	0
*Enterobacter cloacae complex*	17	1	5.9%	0
*Enterococcus faecalis*	27	0	0.0%	0
*Enterococcus faecium*	2	2	100.0%	3.5
*Escherichia coli*	12	4	33.3%	2
*Klebsiella oxytoca*	2	0	0.0%	0
*Klebsiella pneumoniae* ssp. *pneumoniae*	10	9	90.0%	5
*Morganella morganii* ssp. *morganii*	6	0	0.0%	0
*Morganella morganii* ssp. *sibonii*	3	0	0.0%	0
*Proteus hauseri*	1	0	0.0%	1
*Proteus mirabilis*	22	12	54.5%	3
*Pseudomonas aeruginosa*	37	2	5.4%	1
*Serratia liquefaciens group*	1	0	0.0%	0
*Serratia marcescens*	5	0	0.0%	0
*Staphylococcus aureus*	67	34	50.7%	2

**Table 3:** Multivariable logistic regression analyses of factors associated with multidrug resistance. Adjusted odds ratios (aORs) and 95% confidence intervals were estimated using logistic regression with patient-clustered robust standard errors. The primary model included polymicrobial infection, Gram-negative involvement, age and sex. The HbA1c ±30-day model additionally included HbA1c as a continuous variable. MDR, multidrug resistance; HbA1c, glycated hemoglobin; aOR, adjusted odds ratio; CI, confidence interval.

Model	Variable	aOR	95% CI Low	95% CI High	*p*-Value	*N* Episodes	*N* Patients
Primary MDR	Polymicrobial	4.520	1.907	10.716	<0.001	157	135
Primary MDR	Gram-negative	0.378	0.148	0.965	0.042	157	135
Primary MDR	Age (per year)	1.013	0.973	1.054	0.536	157	135
Primary MDR	Male sex	0.819	0.340	1.971	0.656	157	135
HbA1c ±30 days	Polymicrobial	4.038	1.377	11.841	0.011	94	85
HbA1c ±30 days	HbA1c (per 1%)	0.912	0.693	1.200	0.510	94	85
HbA1c ±30 days	Gram-negative	0.391	0.121	1.263	0.117	94	85
HbA1c ±30 days	Age (per year)	1.005	0.958	1.055	0.829	94	85
HbA1c ±30 days	Male sex	1.109	0.313	3.926	0.873	94	85

## Data Availability

The data that support the findings of this study are not openly available due to reasons of sensitivity and are available from the corresponding authors upon reasonable request. Data are located in controlled access data storage at Multimedica IRCCS.
